# Greek medical professionals approaches and understanding of health literacy: a qualitative study

**DOI:** 10.1186/s12913-023-10226-y

**Published:** 2023-11-06

**Authors:** Eleni Louizou, Nikolaos Panagiotou, Εmmanouil Smyrnakis, Savvas Anastasiadis, Konstantinos G. Diamantis, Foivos Papamalis, Panagiotis D. Bamidis

**Affiliations:** 1 Faculty of Health Sciences, School of Medicine, Aristotle University of Thessaloniki (AUTH), Thessaloniki, P.O. Box 376, 54124 Greece; 2 Faculty of Economic and Political Sciences, School of Journalism & Mass Communications, Aristotle University of Thessaloniki (AUTH), Thessaloniki, Greece

**Keywords:** Health literacy, Physicians, Academic, Communication methods, Greece, Qualitative study

## Abstract

**Background:**

Health literacy holds significant importance for medical professionals, as it is widely acknowledged as a key element in enhancing health promotion and overall well-being. The primary objective of this study is to explore Greek physicians’ comprehension of health literacy, the significance they attribute to it, their strategies for addressing patients with low health literacy, and the potential barriers they face while striving to enhance a patient’s health literacy. In this context, we examine the communication methods employed by physicians as an integral part of their approach to improving a patient’s health literacy.

**Methods:**

A qualitative study was conducted between April 29, 2021, and February 17, 2022, utilizing in-depth, semi-structured interviews with 30 Greek medical professionals, of whom 15 were university professors. The research sample selection methodology employed in this study was purposive sampling. Data analysis was conducted using inductive thematic analysis.

**Results:**

The majority of physicians were not familiar with the concept of health literacy. The most significant barriers to the development of health literacy among physicians are a lack of time, issues within the healthcare system, and interference from third parties, although they acknowledge that a significant portion of the responsibility lies with them. Effective communication with patients is important for all physicians, as it plays a crucial role in the therapeutic process. When they realize that their patients are not understanding them, they employ communication methods such as using plain language, providing numerous examples, incorporating visuals like pictures and even using drawings.

**Conclusions:**

The findings of this study underscore the importance of implementing targeted initiatives to promote health literacy within the Greek medical and academic community. Integrating health literacy training for physicians into the educational and training curriculum is essential. To accomplish this goal, it is imperative to first address the shortcomings within the healthcare system and improve the working conditions for physicians.

**Supplementary Information:**

The online version contains supplementary material available at 10.1186/s12913-023-10226-y.

## Introduction

Health literacy (HL) is a term developed in the United States in the 1970s as part of a wider social policy, and it has been evolving ever since, with significant and growing importance to public health and healthcare [1]. The importance of HL has gradually increased in the recent years as it is recognized as a key factor in improving health promotion and well-being.

HL is a multidimensional term. It is linked to literacy and entails people’s knowledge, motivation and competences to access, understand, appraise, and apply health information in order to make judgments and take decisions in everyday life concerning healthcare, disease prevention and health promotion to maintain or improve quality of life during the life course [2]. To that end it is critically important how physicians understand issues of HL and especially how they manage them in relation with their patients.

Most health professionals were unaware of their patients’ low levels of HL was first highlighted, according to the report of the Ad Hoc Committee on Health literacy, appointed by The American Medical Association-AMA το 1997 [3].

According to the systematic bibliographic review we carried out, for the period 01/2009–05/2020, the number of qualitative and mixed studies that have been conducted regarding physician’ perception and understanding of HL are limited [4–9] while in some studies the issue of how physicians handle HL issues is partially examined [10–26]. The methodology of this systematic bibliographic review has been described in detail in a registered protocol through PROSPERO (CRD42020212599).

The same can also be witnessed in Greece where despite the research that has been done on HL issues [27–40] physicians’ perception and understanding of HL is not examined. The only study that partially address the issue based on our research is that of De Wit et al. 2020 [41], that explores the context-specific perspectives of older adults and health professionals on HL in later life in Greece, Hungary, and the Netherlands.

These healthcare professionals worked in older adult communities in various roles, including social workers, nurses, general practitioners, and medical specialists. The Greek sample for this research comprises eleven health professionals, consisting of four physicians and seven others. The study examines their interactions with elderly patients.

The present research attempts for the first time to explore the understanding of HL from Greek physicians, the importance they attach to it, the way they manage a low-HL patient in their daily practice and the possible barriers they encounter when trying to develop a patient’s HL. In this context, the communication approach developed from the Greek physicians is examined as part of the HL approach of the physician towards a patient. Thus, the primary objective of this study is to explore Greek physicians’ comprehension of health literacy and its significance, as well as, strategies they employ for addressing patients with low health literacy, and tackling likely barriers. The findings of the study are expected to highlight the importance of HL as a critical factor for the optimization of health care delivery and a reform that it is needed to be implemented that will have positive impact upon the health workforce in Greece. The present study is part of a wider research that aims to explore, in addition to evaluating the ability of Greek physicians to manage HL issues, the effectiveness of physicians’ communication both with patients and health journalists. Our research maintains a consistent focus on HL throughout its various facets.

## Methods

For the present study, we opted for in-depth interviews as our research method, as it enables us to capture the research sample’s understanding of the issue [42, 43]. The qualitative research was conducted following all necessary procedures to ensure a transparent and rigorous process in both conducting and reporting the results [44–46]. To enhance the trustworthiness of our study and the reported results, we adhered to the consolidated criteria for reporting qualitative research [47].

### Sample

The strategy for selecting the research sample was purposive sampling [48]. In total, 32 physicians were eligible for our study; two declined due to their heavy workloads, while 30 agreed to participate.

Our sample selection criteria excluded psychiatrists due to the unique nature of their patient relationships and pediatricians primarily treating children. Medical students were also excluded, but physicians in specialty training were included. To provide a comprehensive view, half of the physician sample comprises university professors.

The present study is part of a broader research project on HL. We applied an additional criterion to our sample selection process, which involved assessing whether physicians had collaborated with medical journalists prior to the interview to discuss their experiences. In parallel, we conducted interviews with medical journalists.

For the recruitment of physicians, we relied on recommendations from medical journalists who had previously collaborated with these physicians, such as through interviews or other means. This approach was taken to ensure that the selected physicians were well-qualified to provide insights on all aspects of the study. Once a physician recommended by a journalist met the criteria, they were included in our sample. In recruiting medical journalists, we employed a combination of purposive sampling and snowball sampling techniques. Between 29/4/2021 and 17/2/2022, 30 interviews with physicians were carried out. Due to the prevalence of the third wave of the SARS-CoV-2 pandemic in Greece, it was decided for security reasons to conduct the interviews by telephone. The average time of the interviews was 39 min/per interview.

After coding 26 interviews, it became evident that no new codes were emerging, indicating that data saturation had been achieved. This recognition came after consultation with team members, as it was apparent that the data collection process was no longer yielding novel or substantial information [49]. The remaining four interviews were also carried out, as the appointments with the physicians had already been scheduled.

### Interview guide

Τhe semi-structured interview has been selected as the most appropriate method to address the research objectives. This choice was based on its effectiveness in gathering personal perspectives, experiences, and insights related to the subject [50]. To facilitate the qualitative interviews, we developed an interview guide (see Additional file 1) [51, 52]. To ensure the reliability of the interview method, the guide was crafted following the approach outlined by Kallio et al. [53].

In addition to this, we conducted a critical appraisal of existing knowledge through a systematic literature review, which is presented and discussed in this article. The incorporation of prior knowledge helped establish a conceptual foundation for the interviews and significantly contributed to shaping the structure of the interview guide.

A preliminary semi-structured interview guide was formed, consisting of the main questions and follow-up questions. The type of questions are open questions, which leave the respondent free to develop his answer, without pre-determinations.

In the next stage, we refined the interview process by removing unnecessary questions, reordering them for better flow, and enhancing the overall quality of data collection. We used two key techniques for this purpose. First, internal testing was conducted to evaluate the preliminary interview guide. Subsequently, we carried out field testing, which closely simulated the actual interview conditions and provided valuable insights into its execution. During this process, we assessed the effectiveness of the questions and made improvements to follow-up questions. We also adjusted the sequence of questions to ensure practicality and better comprehension for the interviewees. This phase included two pilot interviews with two physicians, from which we gathered feedback and made necessary modifications. Ultimately, these refinements led to the formulation of the final interview guide.

Since it was anticipated that physicians might not be familiar with the term HL, in order to facilitate the interview two definitions [2, 54] were read to all participants after they had previously been asked if they know the term and if they could give an interpretation for it.

The interviews were conducted in a way that facilitated the interviewees, at a day and time that was most convenient for them.

This flexibility gave the interviewees control over scheduling, enabling them to choose an appointment that suited their comfort and ensuring privacy by excluding any other individuals from the room to minimize distractions [55].

The study was approved by the Bioethics committee of the School of Medicine of the Aristotle University of Thessaloniki, Greece (Protocol No. 6331/29.7.2020). Participants, after being informed and agreeing to be part of the study, gave their full consent. On the day of the interview, participants gave their verbal consent for the recording of the interview session. Both procedures (verbal and written consent) was approved by the Bioethics committee of the School of Medicine of the Aristotle University of Thessaloniki, Greece. Each interviewee was informed of the expected duration of the interview. All methods were carried out in accordance with relevant guidelines and regulations.

### Data analysis

All data were audio-recorded, and transcribed verbatim. In addition to the interviewer, three members of the research team (NP, SA, KGD) reviewed transcripts for accuracy.

The method of thematic analysis [56] was chosen for the analysis of the data. This particular method is suitable for qualitative in-depth interviews and makes it possible to analyze human experiences in relation to the topic. Themes emerged directly from the data, without a predetermined coding framework, using inductive analysis. The significance of these themes was not solely determined by quantitative criteria but rather by their ability to encapsulate essential aspects related to the research subject [57, 58].

Initial coding began once the first six interviews were completed. The transcriptions were analyzed independently by three researchers EL, SA, KGD who worked independently on the first six interviews before discussing the preliminary codes and generating initial themes. The consultations continued between the researchers while the coding of the interviews continued to compare the themes identified by each researcher and to establish links among the main themes. To resolve emerging disagreements, a supervisor (NP) was added, and the process was repeated until a strong consensus was reached. The final themes and sub-themes were approved by the team members. Data extraction was performed with QSR NVivo 12 PLUS software.

## Results

The demographic profile of the 30 physicians who participated in the research are presented in Table 1.

**Table 1:** Physicians demographic profile (*n* = 30)

Physicians	
Age in years (average = 56.9)	n (%)
30–45	2 (7)
46–60	16 (53)
61>	12 (40)
Gender	
Female	7 (23)
Male	23 (77)
Medical Specialty by years (average = 25.9)	(*n* = 28)
5–15	3 (10)
16–25	10 (36)
26–35	9 (32)
36–46	6 (22)
Specialization	(*n* = 30)
General Practitioner	6 (20)
Cardiologist	4 (13)
Internist	3 (10)
Pulmonologist	3 (10)
Radiologist	3 (10)
Other^a^	11 (37)
Status	
University Professor	(*n* = 15)
Director of Clinic (Clinicians)	8 (53)
Laboratory Director	3 (20)
Emeritus Professor (ex- Clinician)	1 (7)
No administration position (Clinicians)	3 (20)
NHS	(*n* = 10)
Director at NHS (Clinicians)	9 (90)
No administration position (Clinician)	1(10)
EX – NHS Personnel	(*n* = 3)
Pensioner (ex-Clinician)	2 (67)
Private practice	1 (33)
Clinical Trainee	(*n* = 2)

* Prof.* University Professor, *NHS* National Health System, *GP* General Practitioner

^a^Obstetrician – Gynecologist (*n* = 2) Surgeon (*n* = 2) Anesthesiologist (*n *= 1) Dermatologist (*n* = 1) Nephrologist (*n* = 1) Neurologist (*n* = 1) Orthopedic surgeon (*n* = 1) Otolaryngologist (*n* = 1) Urologist (*n* = 1)

The themes and sub-themes that emerged from the thematic analysis are presented in Table 2.

**Table 2:** Themes and subthemes

Themes	Subthemes
**Perception and management HL**	Definition – interpretation Profile of patients with low HL In his/her own language Use of tools and other communication methods
**Barriers**	Time Systemic factors Attitudes Language Medical Jargon Third Party & Media Interference
**Developing HL as a responsibility**	Responsibility of the physician without the support of the health system The role of the State The role of the media
**The development of HL as a priority**	Benefits Confidence
**Physician Mistakes on H**L	Overestimation of the patient’s HL Working conditions Denial of physicians for error

### Perception and management HL

We began by inquiring whether physicians were acquainted with the term ‘health literacy.‘ The overwhelming majority of physicians, with very few exceptions, indicated that they had never encountered the concept of HL. Even among those who asserted some familiarity with the term, their responses revealed that most had a limited understanding of the concept. When asked to interpret the term, many associated it with cognitive abilities, such as knowledge acquisition, perception, and understanding related to health matters.

However, a very small subset of physicians demonstrated a deeper understanding of the concept of HL. In these instances, some physicians expanded their interpretation beyond cognitive abilities to encompass behavioral aspects. They attributed to individuals a more proactive role, such as *‘the utilization of acquired knowledge*’ or *‘being a proficient user of healthcare services*.‘

Each physician perceives the patient’s level of HL in a different way and attaches different importance to its meaning. The characteristics that make up the profile of a person with low HL, are summarized as follows. Usually, the patient has a low level of education and training, have difficulty communicating and difficulty understanding what the physician says.

Some patients prefer not to be informed about their condition and treatment, while others may harbor doubts or fears related to their physician or feel uncomfortable about asking questions.

Also there is difficulty for these people to follow the treatment. A Professor of Neurology, Director of a University Neurology Clinic states that the patient who has low HL “*has not understood how important it is to take his medicine every day or whenever and to take it on time*” or that he should be consistent with the examination program he should follow, pointing out that they are usually not able to evaluate what the physician tells them.

Almost most physicians when asked how they address a patient with a low level of HL responded that they try to talk to them “*in simple words* and avoid the use of medical jargon. For all physicians it was particularly important to be understood and perceived by their patients. “*The best patient is the informed patien*t” stresses a General Practitioner.

Many physicians mentioned that when they spoke to patients in their “*own language*” they felt they connected better with them.

Physicians recognize that it is especially important that patients fully understand the issue in order to communicate effectively with them. A General Practitioner Director at NHS in day centers points out “*because the procedure at home: ‘take this medicine, so many days, morning-evening, and come back in a month’ is over*”. Therefore, the adaptation of the physicians to the level that they perceive the patient to be in, is the only way for their cooperation with the patient to have a positive outcome. A Professor of Pulmonology and Director of Clinic explains that by the way the patient speaks to him when he asks for certain information, he understands what “*level*” he is at. “*Then I adjust the language I use accordingly*” he concludes.

Many physicians, in order to be understood by their patient, reported that they use examples even from the daily life of the person to whom they are addressing.

Physicians also employ visual aids, including images, to provide patients with a better understanding of the nature of their condition, diagnostic processes, treatment options, and more. They utilize printed materials, such as reputable scientific or pharmaceutical company brochures. Additionally, some physicians emphasized the value of informational videos tailored to specific diseases, such as hypertension and diabetes mellitus, in enhancing patient HL and treatment adherence. A few physicians recommend that patients visit trustworthy health information websites, such as those maintained by European scientific societies.

Three physicians mentioned employing the *‘teach-back method*,‘ wherein they ask the patient to recap what they’ve been told to gauge their level of understanding. Some physicians also noted that they spend additional time with patients who have lower HL as part of their patient management approach. A few physicians mentioned treating patients with low HL in the same manner as other patients, while others emphasized the importance of patient education.

### Barriers

Physicians identified specific barriers that hinder their efforts to enhance their patients’ HL. These barriers include time constraints, systemic issues within the healthcare system, language barriers, the use of medical jargon, patients’ attitudes, deep-seated perceptions, and third-party interference, among others.

The suffocating and stressful time conditions under which physicians work in Greece, combined with the heavy workload they have to manage every day, is a barrier that prevents a physician from developing a patient’s HL. “*The lack of time, our multiple preoccupations with different subjects at the same time, e.g. teaching for physicians that are also University Professors, research and at the same time providing health*” burden the physician, as explained by a Director of a University. The time he can allocate to a patient is not what is required, as a Professor of Neurology, Director of a University Clinic argues: “*in order to see the patient holistically, to evaluate him, we must allocate at least 50 mins of the hour with him*”. Factors originating from malfunctions of the health system such as the lack of staff, the work pressure due to the large flow of patients and the stress created for the physician by the patients staying in the waiting area, the non-specialization of the nursing staff, etc., burden in addition to physicians in their work and hinder those who wish to develop the HL of their patients. The pandemic has exhausted some physicians even more to the point that they consider the availability of time as a “*great luxury*” according to an Anesthesiologist Director at NHS.

Physicians face an additional barrier when attempting to improve their patients’ HL: the attitudes they encounter, which may include ‘*stereotypes*’ related to cultural, religious beliefs, or even ‘*fanaticism*,‘ ‘*ideologies*,‘ and more. Furthermore, patients’ personal beliefs and ‘*entrenched*’ perspectives, whether concerning the illness itself or its treatment, can also present significant barriers, as pointed out by a Cardiologist specializing in a University Cardiology Clinic. A General Practitioner adds.“*There are really patients to whom no matter what you say, no matter how many times you’ve said it, it still doesn’t go away, there’s no change in their attitude and behavior*,“ he explains. Some of the physicians examined argued that often these attitudes come from specific social groups, such as the Roma.

Some physicians reported that communicating in a foreign language is sometimes a barrier to working with patients, especially in recent years as withpatients coming from third countries, refugees, etc. A Director of Radiodiagnostics of the National Health Service characteristically mentions “*Many times we are using body language in order for the other person understand what we are asking fo*r”. The use of a translator to overcome the language barrier involves a degree of difficulty and many times this role is taken over by a relative husband who, as explained by a Professor of Obstetrics - Gynecology, Director of the University Clinic “*we don’t know exactly what he/she is translating and whether he/she will translate correctly*”.

The popularization of medical jargon by the physician to make it understandable by the patient it is difficult or tiring for some physicians because, as one university physician explained, *in this way physicians “has learned to read, pronounce, deliver to students and to use for decades*”.

Third-party interference refers to the barriers faced by physicians when patients use what appears to be “medical information” their health situation, which may come from the patient’s direct or indirect environment or even from the media. “*When we have a patient, we should be very worried, who will he meet when leaving the physician’s office, what will the pharmacist say to him, if he goes to another physician who may have a different perception, possibly wrong, what will he say to him his family, what one, the other will say to him, etc*” explains a university physician.

The way patients manage information received from sources other than their physician, including the media, leads to a unique group of patients who exhibit *‘pseudo-inflated literacy*.‘ These individuals often believe they possess a deeper understanding than they actually do, attempting to *‘appear knowledgeable*’ explains a Radiologist Director at NHS Hospital.

The patient’s family, relatives, friends, and the surrounding environment can be considered an interference or even a barrier for some physicians. A resident Cardiologist at a University Cardiology Clinic often encounters situations where patients mention, “*So my relative who did this told me.*.“

On the other hand, three professors reported that they do not encounter significant barriers or major issues when attempting to enhance a patient’s HL.

### The development of health literacy as a responsibility

Almost all physicians recognize the important part of responsibility they themselves have to a significant degree in developing patients’ HL. In fact, many physicians find that the responsibility lies exclusively with them, as the conditions in Greece have been shaped, with the way the National Health System operates. In other countries, such as the USA, the United Kingdom, in addition to the important role played by the state itself in matters of HL of citizens, the physician is supported by a team of health professionals, such as high-level nursing staff, who shoulder some of the responsibilities of a physician. “*They all form a team and play a role. Everyone in their own separate role but where they are equally*” as an Internist explains.

A General Practitioner, Director at NHS in a Regional Clinic clarifies “*But of course the health system structure is mostly responsible to create the conditions that allow the patient to come prepared and knowledgeable or the equivalent to the physician in a way to compel him to do his job and to ensure the conditions under which he will do it*”.

The insufficient role of the State in Greece in the development of the HL of citizens was mentioned by many physicians. An ENT University Clinic Director, without sidelining the physician’s responsibility clarifies that the development of HL at a collective level is the responsibility of both the state and society: “*Health education, which does not exist at the moment, is a main agent of health awareness which is currently lacking at the state level. There is prevention, but there is no health treatment*”.

The role of mass media in the development of HL was highlighted as decisive by some physicians. Few physicians argued that the responsibility for developing patient HL should rest solely with the physician, primarily because of the physician’s “*inseparable relationship*” with the patient.

### The development of health literacy as a priority

Almost all physicians unequivocally and categorically affirmed that prioritizing the development of their patients’ HL is of utmost importance to them. Physicians recognize that enhancing a patient’s HL holds significant importance, as it contributes to improved disease management for both the physician and the patient, particularly in the case of chronic diseases.

“*It is important to educate the patient*” points out a university Cardiologist, while a General Practitioner emphasizes that there will also be benefits for the physicians themselves, as it will help them more to draw useful conclusions for the patient. The development of the patient’s HL, according to some physicians, helps to create a relationship of trust between the physician and the patient. A General Surgeon Director at a University Clinic explains that it is a priority because it facilitates dialogue, information and participation of the patient in the overall effort of diagnosis and treatment, as he underlines *“When he understands what is happening and why it is happening and what will happen, then it is very logical that the participation of each person should be much more active*”. 

### Physicians’ mistakes on health literacy

Most physicians admitted that they may have misjudged a patient’s level of HL. Only six physicians mentioned that they have never made a mistake about a patients level of HL. A common mistake that physicians seem to make is that they may overestimate a patient’s HL. This may occur mainly at the beginning of the physician’s relationship with the patient. “*Many people can be very good at hiding whether they understand or not*” explains a University Obstetrician-Gynecologist. A Professor of Cardiology, Director of a University Clinic explains that the mistake lies in the fact that many patients appear to be very well informed “*but in the end it’s a lot worse than you think. As a result, you talk on a different level and at some point you realize that instead of helping them, you have confused them even more*”.

Several of the factors mentioned by physicians that prevent them from developing patients’ HL also contribute to the misestimation of the level of HL, such as time pressure, health system malfunctions, workload and the work fatigue of the physician etc. “*When I finish my working hours, that I have seen a lot of patients, e.g. 20–40, I feel that I might have made some mistakes due to the limited time that I can devote to each patient”* explains a Director of the University Pulmonology Clinic. Two physicians referred to the need to educate physicians to avoid misjudging the level of HL of their patients.

There were six physicians who claimed that they have never made a mistake in assessing the level of HL of their patients. An experienced university Internist maintains that no general mistakes are made in this part because it does not involve a degree of difficulty for physicians “*Perceiving the level of literacy is not difficult. I don’t think there are any mistakes in it, no*”. The other physicians cited their work experience, denying any wrongdoing.

## Discussion

As it results from our study, it was found that the concept of HL has possibly not been introduced appropriately to medical professionals in Greece. This is evident from the fact that most Greek physicians who participated in the research, were not familiarized with the concept. The fact that half of the sample consisted of Greek university medical professors may mean that the concept has not been properly introduced in the Greek academic environment either. Physicians do not seem to aware that the communication methods and tools they use to make themselves understood by patients who have difficulty understanding medical information are related to HL. Nevertheless, once the term was given to the physicians for the research needs, almost all physicians recognized its importance and argued that it would be a priority for them to develop the HL of their patients. The unfamiliarity of the concept HL among Greek physicians is in line with the findings from international literature which certify the low level of knowledge that health professionals have about HL [6, 59–62].

According to Greek physicians, some of the characteristics that make up the profile of a patient with low HL can be found in the study by Smith et al. 2014 [9]. In this study, some physicians asserted that patients with low HL exhibited a paternalistic behavior of the type *‘I do not want to know*.‘ In the study by Khuu et al. 2016 [5], physicians observed that some patients with low HL have doubts or fears regarding their physicians.

The difficulty for individuals with low HL in adhering to treatment and medication regimens is noted in several studies, where physicians have asserted that patients with low HL encounter challenges in understanding prescriptions and managing their medications [6, 9, 10, 22]. They often lack the ability to organize themselves [9, 12] or have a poor understanding of the available resources or treatments [13].

Conversely, a component of HL related to the patient’s ability to navigate and utilize the healthcare system, where individuals with low HL faced the most difficulties, was mentioned by physicians in other studies [5, 6, 9, 10, 13, 15]. However, it was not explicitly mentioned by all the Greek physicians in our study.

Some of the communication methods employed by Greek physicians align with those suggested in other studies on the topic, such as repetition [23] or using straightforward language, like the *‘teach-back method*’ [9].

When it comes to the barriers that physicians face in developing HL, the lack of available time is a significant factor consistently highlighted in qualitative and mixed studies [4, 6, 9, 12, 14, 15, 20, 21, 24, 25]. Physicians often mention time constraints, leading to limited availability for patients with low HL [4, 12, 14, 15, 24, 25]. In one study, it was noted that spending sufficient time with patients was essential, even for those with high levels of HL [9]. The constraint on time can potentially lead to physicians misjudging their patients’ HL levels [63].

Physicians argue that barriers stemming from the dysfunction of the healthcare system, such as staff shortages, a high number of patients, and work-related stress, burden them and hinder their efforts to develop HL for their patients [4, 6, 7, 15, 25, 59, 61, 64, 65].

This finding is also consistent with the research of De Wit et al. 2020 [41] which argues that in Greece, barriers encountered in clinical environments further burden the work and commitment of healthcare professionals. The suffocating and stressful work conditions experienced by Greek physicians are not unique and have also been reported in other countries, where high levels of burnout among doctors have been documented, with implications for the well-being and retention of healthcare professionals as well as the quality of patient care [66–70].

One crucial factor emerging from our study is the influence of third parties, which appears to have a significant impact on the physician-patient relationship. Barriers mentioned by Greek physicians regarding their communication with patients, stemming from the patient’s environment or information found on the internet, align with findings from qualitative/mixed studies in the international literature [7, 9, 11, 20, 22, 25]. Greek physicians reported language barriers less frequently compared to those documented in international literature [4, 6, 9, 10, 13, 23–25].

The barrier posed by the use of medical jargon is present in some studies, highlighting how medical terminology can impede certain physicians’ communication with patients and consequently hinder the development of HL [8, 11, 13, 14, 20]. Barriers related to attitudes tied to *‘cultural or religious beliefs*’ are encountered in numerous studies and are among the major obstacles faced by physicians in developing HL [4–6, 8, 10, 13, 14, 19, 24, 25].

Regarding the barriers that limit physicians to developing HL, no physician reported a lack of knowledge about HL despite the fact that our research actually revealed substantial unfamiliarities with the term. Nevertheless, most physicians admitted in their interviews that they may have misjudged a patient’s level of HL. Overestimating a patient HL level is among the biggest mistakes, as it has been mentioned that Greek physicians can make. This mistake aligns with the findings of other studies as mentioned in the literature [7, 63, 71–73].

Almost all Greek physicians unequivocally and categorically stated that prioritizing the development of their patients’ HL is essential. They also recognize the significant part of the responsibility they themselves bear in developing patients’ HL. This is a crucial and clear finding that is not often encountered in other studies. In one study addressing the issue of responsibility in HL, some physicians appeared willing to assume responsibility towards their patients [7]. In another study, physicians were more inclined to consider the lack of HL as a ‘*deficiency*’ of the patient rather than a responsibility of the physician to develop patient HL [6].

Greek physicians asserted that HL is a priority because they believe it helps establish a relationship of trust with the patient. It seems that there is a link between HL and trust in the physician-patient relationship [18, 74, 75]. People with low HL were more likely to be distrustful of the physician [76], particularly citizens who belong to a different nationality from the country where they reside [5, 10, 13, 24, 25].

In some studies that exclusively explore the perspective of physicians on HL issues, we find certain themes similar to our own. The study by Lambert et al. 2014 [6], for instance, identifies similar barriers, such as systematic/structural factors, time constraints, social and cultural factors, as well as the theme related to health professionals’ perception of HL.

In Hughson et al. 2018 [4], similar emergent themes were cultural barriers and time constraints. Sadeghi et al. 2013 [8], in addition to cultural barriers, also includes language barriers among them. Salter et al. 2014 [7], is the only one where a theme of responsibility in HL emerged. Finally, in Smith et al. 2014 [9], physicians identified a patient’s literacy level as a theme where they described how they perceive patients’ HL levels and their characteristics accordingly. In another theme in the same study, challenges and strategies for communicating concepts to patients with low HL are described.

HL measurement tools [77–80] could serve as valuable aids for physicians. Directly identifying a patient’s HL level using one of the available tools would assist healthcare professionals in tailoring their communication to each individual’s specific needs, potentially leading to more effective physician-patient communication. However, using these tools requires communication training for physicians to develop patients’ HL, which could significantly enhance patient communication and improve health outcomes [81]. Nevertheless, only a few physicians mentioned the necessity of educating physicians to prevent misjudging their patients’ HL levels and to determine how they can contribute to its development. There appears to be a lack of training and education, especially in the area of patient-centered communication and HL-related skills. This raises a concern and underscores the importance of considering this issue when modernizing medical curricula.

Our study has some limitations. When Greek physicians were initially asked if they had ever heard of the concept of HL, the vast majority responded negatively. The fact that the concept had to be explained to the physicians in order to complete the interview could be a limitation for the answers provided thereafter. Nevertheless, through the responses provided by the physicians, it was evident that HL is a part of their daily practice. The second limitation is related to the participant selection, which was carried out using purposive sampling. We attempted to minimize this limitation by establishing strict inclusion and exclusion criteria in the sample selection process, following a transparent and rigorous methodology. The third limitation arises from the qualitative nature of the research and its focus on a specific professional field. As such, the findings cannot be generalized to other professional domains or to the general population in Greece.

## Conclusions

Our research highlights that Greek physicians are not appropriately familiarized with the concept of HL. The biggest problems they face when they try to develop patients’ HL results from the lack of time, malfunctions of the health system, work pressure, perceptions of patients, while third-party interference become particularly important as they affect physician-patient communication.

The development of a patient’s HL is recognized by almost all physicians as an important responsibility and priority for a physician. At the same time, however, the physicians argue that the way the Greek health system works today, HL is degraded by the system itself and its way of functioning, as well as by the fact that the responsibility for its development rests exclusively on the physician and is not shared with anyone else, both inside and outside the health system. The demanding working conditions for physicians in Greece also seem to be the biggest cause that can lead a physician to misjudge a patient’s HL. This results in most of the time physicians overestimating the patient’s HL.

The potential introduction of HL into the Greek healthcare environment may face challenges unless the dysfunctions within the health system and the challenging working conditions of healthcare professionals are addressed as a priority. There is a need to explore ways in which the Greek healthcare system can provide more support to healthcare professionals and implement targeted interventions promptly. Concurrently, integrating HL into the Greek academic environment and incorporating HL-related training into the official medical curriculum is crucial. Greek universities and relevant policymakers should consider updating medical definitions and terminology to align with recent developments in the field of medicine.

The results of this study highlight the need for further research in Greece regarding HL. The HL levels of the Greek population should be part of this research since patients with low HL face more difficulties in understanding medical information and communicating with the physician, parameters that contribute decisively to their good health outcome.

### Supplementary Information

**Additional file 1.** Interview Guide; An interview guide was created in order to conduct the qualitative interviews.

## Data Availability

The datasets generated and/or analyzed during the current study are not publicly available due to potential privacy violation reasons but are available from the corresponding author on reasonable request.

## Abbreviations

HL: Health Literacy
NHS: National Health System
