# Supplementary material for: Greek medical professionals approaches and understanding of health literacy: a qualitative study
Source: BMC Health Serv Res. 2023 Nov 6;23:1209. doi: 10.1186/s12913-023-10226-y (PMC10626757; doi:10.1186/s12913-023-10226-y)
Supplement: Supplementary file 1 — Additional file 1. Interview Guide; An interview guide was created in order to conduct the qualitative interviews. [file 12913_2023_10226_MOESM1_ESM.pdf]

Additional file 1. Interview guide.

Topic (a): How doctors conceptually approach health literacy

-Have you ever heard the term “health literacy”?

-If so, how would you define health literacy?

At this point to facilitate the interview two definitions of HL are read to the interviewee.

a) Health Literacy entails people's knowledge, motivation and competences to access, understand, appraise, and apply health information in order to make judgments and take decisions in everyday life concerning healthcare, disease prevention and health promotion to maintain or improve quality of life during the life course. [1]

b) Health literacy represents the cognitive and social skills which determine the motivation and ability of individuals to gain access to, understand and use information in ways which promote and maintain good health. [2]

Topic (b): How do doctors manage health literacy in relation to their patients

-How do you manage a patient with low health literacy?

-When your patients don't understand what you tell them what do you do?

-Do you have a way of checking/checking that your patients have fully understood what you are telling them?

-Is there any other communication method you use to simplify and explain health information to ensure patient understanding?

If yes/no: why?

-Are there barriers you encounter when trying to develop your patient's health literacy?

Can you name specific obstacles-problems?

-Despite the barriers you encounter, is it a priority for you to develop the patient's health literacy?

If yes/no: why?

-Do you think it is the doctor's responsibility to develop the patient's health literacy?

If yes/no: why?

Who could be responsible for this?

If so, who?

-Do you think you may have ever misjudged a patient's level of health literacy?

If yes/no: why?

References

1. Sørensen K, Van Den Broucke S, Fullam J, Doyle G, Pelikan J, Slonska Z, et al. Health literacy and public health: A systematic review and integration of definitions and models. BMC Public Health. 2012;12(1):80.

2. Nutbeam, D. Health promotion glossary. Health Promot Int. 1998;13(4):349–64.
